# RIPK1-dependent cell death: a novel target of the Aurora kinase inhibitor Tozasertib (VX-680)

**DOI:** 10.1038/s41419-017-0245-7

**Published:** 2018-02-12

**Authors:** Sofie Martens, Vera Goossens, Lars Devisscher, Sam Hofmans, Polien Claeys, Marnik Vuylsteke, Nozomi Takahashi, Koen Augustyns, Peter Vandenabeele

**Affiliations:** 1 Inflammation Research Center (IRC), VIB, Ghent, 9052 Belgium; 2 Department of Biomedical Molecular Biology (DBMB), Ghent University, Ghent, 9052 Belgium; 3 Laboratory of Medicinal Chemistry, University of Antwerp, Antwerp, 2610 Belgium; 4 Gnomixx, Melle, 9090 Belgium

## Abstract

The Aurora kinase family (Aurora A, B and C) are crucial regulators of several mitotic events, including cytokinesis. Increased expression of these kinases is associated with tumorigenesis and several compounds targeting Aurora kinase are under evaluation in clinical trials (a.o. AT9283, AZD1152, Danusertib, MLN8054). Here, we demonstrate that the pan-Aurora kinase inhibitor Tozasertib (VX-680 and MK-0457) not only causes cytokinesis defects through Aurora kinase inhibition, but is also a potent inhibitor of necroptosis, a cell death process regulated and executed by the RIPK1, RIPK3 and MLKL signalling axis. Tozasertib’s potency to inhibit RIPK1-dependent necroptosis and to block cytokinesis in cells is in the same concentration range, with an IC50 of 1.06 µM and 0.554 µM, respectively. A structure activity relationship (SAR) analysis of 67 Tozasertib analogues, modified at 4 different positions, allowed the identification of analogues that showed increased specificity for either cytokinesis inhibition or for necroptosis inhibition, reflecting more specific inhibition of Aurora kinase or RIPK1, respectively. These results also suggested that RIPK1 and Aurora kinases are functionally non-interacting targets of Tozasertib and its analogues. Indeed, more specific Aurora kinase inhibitors did not show any effect in necroptosis and Necrostatin-1s treatment did not result in cytokinesis defects, demonstrating that both cellular processes are not interrelated. Finally, Tozasertib inhibited recombinant human RIPK1, human Aurora A and human Aurora B kinase activity, but not RIPK3. The potency ranking of the newly derived Tozasertib analogues and their specificity profile, as observed in cellular assays, coincide with ADP-Glo recombinant kinase activity assays. Overall, we show that Tozasertib not only targets Aurora kinases but also RIPK1 independently, and that we could generate analogues with increased selectivity to RIPK1 or Aurora kinases, respectively.

## Introduction

Mitosis is a multi-step process that is tightly regulated by several classes of kinases, like cyclin-dependent kinases (CDKs) and Aurora kinases^1^. The Aurora serine/threonine kinase family consists of three kinases in mammals: Aurora A, Aurora B and Aurora C^2^. All three Aurora kinases, although being structurally similar, have different functions and cellular localisations during mitosis^2–5^. Aurora A and B are expressed in most cell types and play important roles in centrosome maturation, mitotic spindle formation, kinetochore assembly and cytokinesis, the final step of cell division^2,6,7^. Aurora C, in contrast, is only expressed in testis where it is crucial for spermatogenesis^2^. Aurora A and B have been described as oncogenes and increased expression or polymorphisms of these kinases have been observed in several types of cancer^6^, like breast cancer^8,9^, prostate cancer^10,11^ and non-small-cell lung carcinoma^12^. Inhibition of Aurora kinase results in failure of G2/M transition, abnormal spindle formation leading to cytokinesis defects and apoptosis^13^. Several Aurora kinase inhibitors have been developed, like MLN8054 and MLN8273, which are currently in clinical trials phase I and phase II for the treatment of solid tumours and hematopoietic cancers^5,6^. In this paper, we will focus on the pan-Aurora kinase inhibitor Tozasertib (VX-680, MK-0457), which has been described as a type I small molecule inhibitor that targets the ATP-binding pocket of Aurora kinases^14^. Tozasertib retards tumour growth in xenograft models (a.o. HL-60 and HCT116)^2,3,15^ and was in clinical trial phase II for solid tumours and leukaemia^5,6^. Although Tozasertib treatment has clear anti-tumour activity, studies were discontinued due to toxic adverse effects^5,16^. In a broad spectrum drug/kinome-binding study, it was reported that Tozasertib can bind to receptor-interacting-protein kinase 1 (RIPK1) with a *K*_d_ of 20 nM^17^, while the *K*_d_ value is 0.6 nM for binding to Aurora A^15^. RIPK1 is part of the receptor-interacting-protein kinase family that is involved in the regulation of cell death processes and inflammation^18–21^. Reports are also emerging of its involvement in tumorigenesis^22,23^. Necroptosis is a cell death process regulated by a kinase signalling cascade in the absence of caspase activation^18,21,24–26^. TNF-induced necroptosis involves activation of RIPK1 and RIPK3 in a necrosome complex, leading to the activation of the execution protein-mixed lineage kinase domain-like protein (MLKL)^18,20,27–29^. Here, we characterise Tozasertib as cytokinesis and necroptosis inhibitor in a cellular model system, simultaneously analysing multiple image-based parameters reflecting these phenotypes. A screening of 67 novel Tozasertib analogues suggests that both pathways are independent from each other and that analogues can be identified that have a higher selectivity for either cellular response.

## Results

### Tozasertib induces cytokinesis defects and inhibits necroptosis at similar dose dependency

**Fig. 1:**
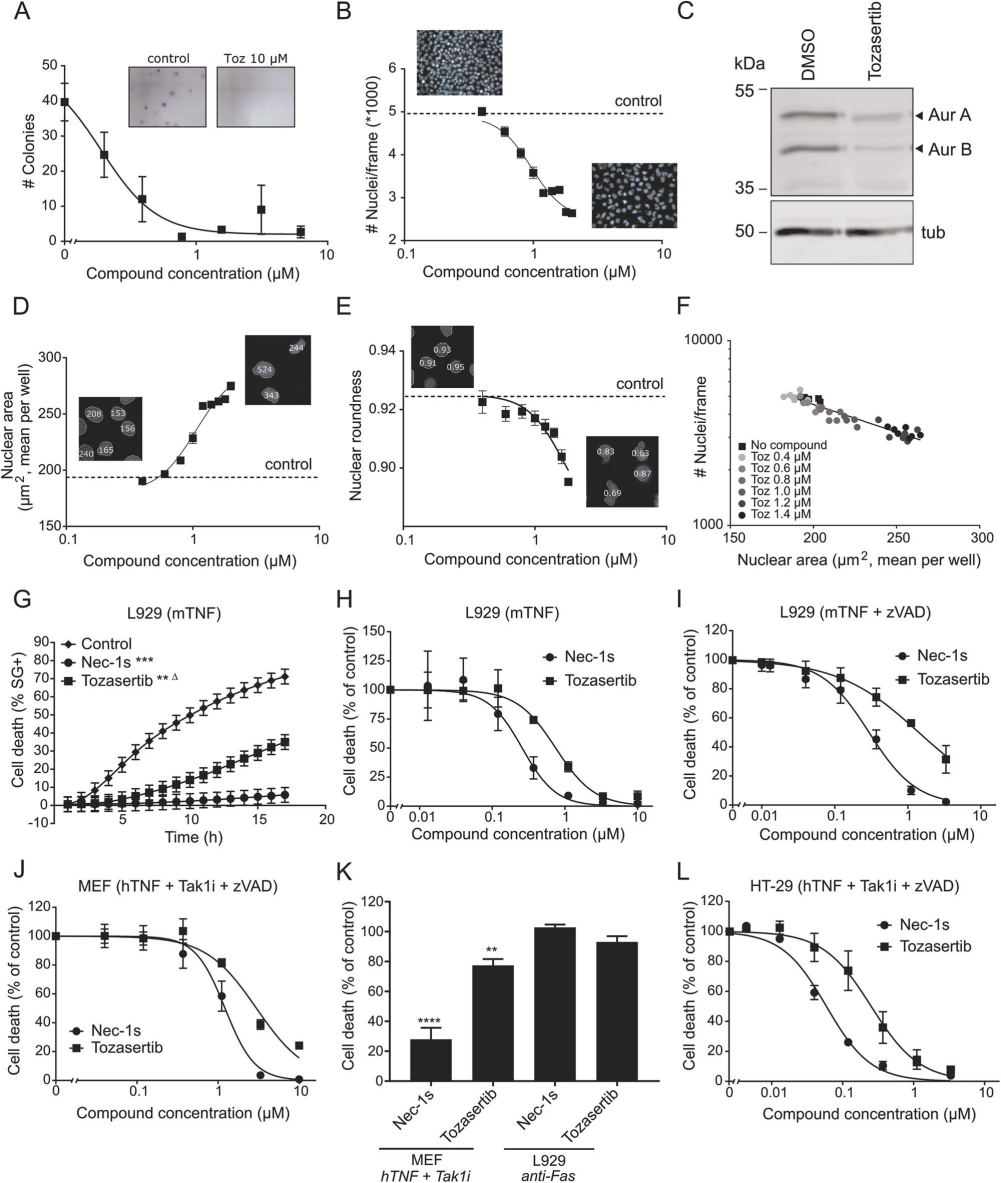
Tozasertib induces cytokinesis defects and inhibits necroptosis with similar dose dependency. **a** L929sAhFas cells were treated with Tozasertib (concentration as indicated) for 24 h. Then, a clonogenic assay was performed to determine colony-forming capacity. Quantification was performed using ImageJ. Data represent mean values ±S.E.M. (*n* = 3). **b**,** d-f** L929sAhFas cells were treated with DMSO (control) or Tozasertib (Toz) (concentrations as indicated) for 24 h (**f**) or 48 h (**b**, **d**–**e**). Cells were stained with Hoechst (1 µM) and high-content images were acquired using BD pathway Bio-imager and analysed using Columbus^TM^ software. The dotted control line indicates the real *y*-axis value corresponding to a normal nucleus (DMSO treatment). Data represent mean values ±S.E.M., *n* = 6. Nuclear roundness is quantified as with 0 being a straight line and 1 being a perfect circle. Number of nuclei was plotted against the nuclear area (µm^2^) for all replicates (*n* = 6). Linear relation between parameters was shown using GraphPad Prism 7. **c** L929sAhFas cells were treated with DMSO or Tozasertib (10 µM) for 16 h, lysed and immunoblotted with the indicated antibodies. **g**–**I** L929sAhFas cells were pre-treated with DMSO (control), Nec1s (3 µM) or Tozasertib (3 µM) (**g**) or with concentrations as indicated (**h-i**) for 1 h, followed by mTNF (20 ng/mL) (**g**,** h**) (*n* = 4) or mTNF (20 ng/mL) + zVAD.fmk (20 µM) (**i**) stimulation (*n* = 3). * = Significance compared to DMSO, Δ = significance compared to Nec-1s. (see 'Material and methods' for statistical analysis) **j** MEF cells were pre-treated with DMSO, Nec-1s or Tozasertib (concentration as indicated) for 1 h, followed by stimulation with hTNF (20 ng/mL) + Tak1i (1 µM) + zVAD.fmk (20 µM) for 4 h (*n* = 3). **k** MEF (*n* = 3) and L929sAhFas (*n* = 3) cells were pre-treated with DMSO (control), Nec1s (10 µM) or Tozasertib (10 µM) for 1 h, followed by stimulation with agonistic anti-Fas antibody (250 ng/mL) or hTNF (20 ng/mL) + Tak1i (1 µM), respectively. One-way ANOVA was performed using Tukey correction for multiple comparison (compared to DMSO control). **l** HT-29 cells were pre-treated with DMSO, Nec1s or Tozasertib (concentration as indicated) for 1 h, followed by stimulation with hTNF (100 ng/mL) + Tak1i (1 µM) + zVAD.fmk (20 µM) for 16 h. **g**,** l** Cells were stained with SytoxGreen (5 µM) and cell death was followed over time using the FluoStar fluorescence detection system. **h–k** Cells were stained with propidium iodide (PI) (3 µM) and Hoechst (1 µM) for image analysis with BD pathway. Cell death percentage (% PI-positive nuclei) was calculated as percent of control. Data represent mean values ±S.E.M. (**g–l**). The time kinetic experiment (**g**) was statistically analysed using repeated measurements data analysis and dose responses (IC50 determination) analysed using probit analysis (see 'Material and methods')

Since Tozasertib is a pan-Aurora kinase inhibitor^14^, we used the well-established L929 cellular model for TNF-induced necroptosis research^30^ to confirm inhibitory effects of Tozasertib on cell growth and cytokinesis and to evaluate its effect on cell death. Tozasertib is not toxic on itself in murine L929sAhFas cells at 10 µM or lower (Supplementary Figure 1B), but impairs colony formation in a clonogenic assay from 0.2 µM on (Fig. 1a), illustrating its cytostatic activity. Also, the total number of nuclei per image frame decreased with increasing concentration of Tozasertib (Fig. 1b), reflecting Tozasertib-induced growth arrest (IC50 value of 0.122 µM). Tozasertib-induced inhibition of autophosphorylation of endogenous Aurora A (Aur A) and B (Aur B) confirms that both Aurora kinases are targeted in L929sAhFas cells (Fig. 1c). Using high-content imaging, not only the number of nuclei, but also the nuclear morphology can be assessed^31^. Both nuclear area and nuclear roundness were analysed as parameter for cytokinesis defects (Fig. 1d–e). With increasing concentration of Tozasertib, nuclear area increased (mean increase to 250 µm^2^ at highest concentration) and nuclear roundness decreased (mean decrease to 0.80) (Fig. 1d–e). The parameter nuclear area showed a linear correlation with the number of cells or cell growth in function of the concentration of Tozasertib (Fig. 1f). Therefore, cell number and nuclear area will be further used to evaluate cytokinesis inhibition, a consequence of Aurora kinase inhibition^5–7^ (Fig. 1f). Next, Tozasertib was tested in murine (L929sAhFas, MEF) and human (HT29) cells for inhibition of RIPK1-dependent necroptosis. Indeed, Tozasertib (3 µM) protects against TNF-induced RIPK1-dependent necroptosis over time in L929sAhFas cells (Fig. 1g). This protection is dose dependent with an IC50 value of 0.55 µM (Fig. 1h and Table 1). Although Necrostatin-1s (Nec1s) protects against RIPK1-dependent cell death (Fig. 1g–l), RIPK1 inhibition by Nec1s does not result in cytokinesis defects (Supplementary Figure 2). Also, treatment with GSK’963, a more potent and selective RIPK1 inhibitor than Nec-1s^32^, did not affect cytokinesis in L929sAhFas cells (Supplementary Figure 2), suggesting that RIPK1 kinase activity is not involved in cytokinesis. In sensitising conditions in L929sAhFas cells (mTNF+zVAD.fmk) and in MEF cells, Tozasertib protects against cell death with an IC50 of 1.08 µM (Fig. 1i and Table 1) and 2.56 µM (Fig. 1j and Table 1), respectively. Although Tozasertib inhibits RIPK1-dependent apoptosis in MEF cells, its inhibition on RIPK1-dependent necroptosis in the same cells is remarkably less effective (Fig. 1j, k). This suggests that RIPK1 kinase engagement might be different in apoptotic and necroptotic conditions. In the case of induction of RIPK1-independent apoptosis in L929sAhFas cells by agonistic anti-Fas antibody, no inhibition is observed (Fig. 1k). This indicates that Tozasertib does not affect Fas-mediated apoptosis at the level of FADD/caspase-8 activation, cytochrome *c* release or the Apaf-1-mediated caspase cascade. Finally, Tozasertib also blocks RIPK1-dependent necroptosis in the human HT29 cell line with an IC50 of 0.26 µM (Fig. 1l and Table 1). Both in sensitising necroptosis conditions in L929sAhFas cells (mTNF+zVAD.fmk) (Supplementary Figure 3A) and in HT29 cells (hTNF+Tak1i+zVAD.fmk) (Supplementary Figure 3B), the protective effect of Tozasertib is partially lost at 10 µM and was excluded for IC50 determination. Overall, Tozasertib-induced growth arrest (IC50 0.97 µM), cytokinesis inhibition (IC50 1.06 µM) and necroptosis inhibition (IC50 0.55 µM) show similar dose dependency in different cellular models (Table 1).

**Table 1:** Tozasertib induces cytokinesis defects and inhibits necroptosis with similar potency, while Nec1s inhibits necroptosis without affecting cytokinesis

				Tozasertib		Nec-1s	
		Compound (pre)treatment time (h)	Necroptosis treatment time (h)	IC50 (µM)	95% CI (µM)	IC50 (µM)	95% CI (µM)
Cytokinesis	Clonogenicity	24	–	0.122	0.104–0.139	NT	NT
	Cell growth	48	–	0.969	0.915–1.025	NC	NC
	Nuclear area^!^	48	–	1.056	1.013–1.101	NC	NC
	Nuclear roundness	48	–	1.639	1.521–1.842	NC	NC
Necroptosis	L929-mTNF	1	5	0.554^ΔΔΔ^	0.483–0.635	0.149	0.126–0.174
	L929-mTNF+zVAD	1	3	1.082^ΔΔΔ^	0.923–1.277	0.24	0.210–0.275
	MEF-hTNF+TAK1i+zVAD	1	4	2.561^ΔΔΔ^	2.237–2.934	0.803	0.703–0.914
	HT29-hTNF+TAK1i+zVAD	1	16	0.257^ΔΔΔ^	0.227–0.291	0.077	0.067–0.090

Characterisation and quantification of Tozasertib and Nec1s affecting cytokinesis (clonogenicity, cell growth, nuclear area and nuclear roundness as parameters) and necroptosis (L929, MEF and HT29 cell lines as model systems). IC50 values were calculated and compared using dose-response curves of three independent experiments and Probit analysis (see 'Material and methods'). The 95% confidence intervals (CI) for IC50s are also indicated

*NT * not tested, *NC *  not calculable *!* values represent EC50, *Δ* refers to the significance of the difference relative to the IC50 of Nec-1s

### Tozasertib-induced cytokinesis defects and necroptosis inhibition are not correlated

Previous results with Nec1s and GSK’963 suggested that RIPK1 is not involved in cytokinesis (Supplementary Figure 2). In order to further investigate whether Aurora kinase-dependent cytokinesis and RIPK1-dependent necroptosis are interrelated, a small panel of pan-Aurora kinase inhibitors were tested for their ability to inhibit necroptosis in L929sAhFas cells (Table 2). This panel included Tozasertib, Barasertib (more specific for Aurora B), AMG-900, Danusertib and SNS-314 mesylate. Of all Aurora kinase inhibitors tested, only Tozasertib and SNS-314 mesylate were able to inhibit necroptosis with IC50 values of 1.1 µM and 0.4 µM, respectively (Table 2). AMG-900 inhibits cytokinesis with an IC50 in nanomolar range (nuclear area) but cannot inhibit necroptosis (Table 2). These results suggest that cytokinesis and necroptosis are independent processes.

**Table 2:** Tozasertib-induced cytokinesis defects and necroptosis inhibition are not correlated

Inhibitor	Target	Cytokinesis		Necroptosis	
		EC50	95% CI for EC50	IC50	95% CI for IC50
Nec-1s	RIPK1	NC	NC	0.367	0.187–0.739
Tozasertib	pan-Aurora	0.591	0.230–1.581	1.143	1.405–7.749
AMG-900	pan-Aurora	0.018	0.006–0.093	NC	NC
Barasertib	Aurora B	1.264	0.444–3.181	NC	NC
Danusertib	pan-Aurora	2.528	1.143–4.223	13.92	6.088–47.84
SNS-314	pan-Aurora	0.073	0.018–0.320	0.399	0.129–1.424

L929sAhFas cells were pre-treated with the indicated Aurora inhibitors (dose-response from 10 µM–0.0022 µM) for 1 h, followed by stimulation with mTNF (20 ng/mL) for 5 h. EC50 and IC50 values (µM) were calculated for, respectively, nuclear area and inhibition of necroptosis using nonlinear regression in GraphPad Prism 7. The 95% confidence intervals (CI) for IC50s are also indicated

*NC* not calculable

### Dissection of cytokinesis inhibition and necroptosis inhibition by SAR of Tozasertib analogues

**Fig. 2:**
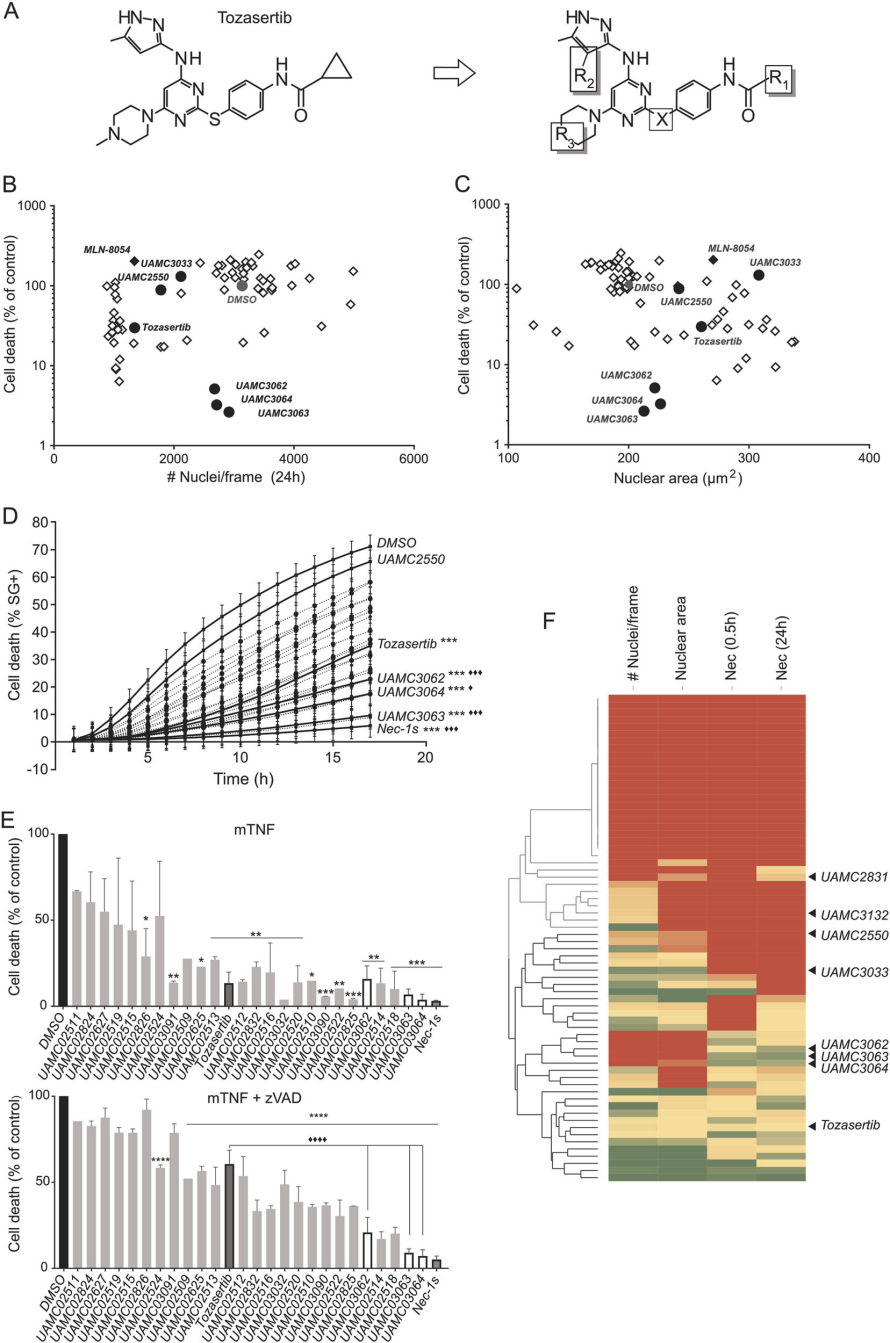
Dissection of cytokinesis inhibition and necroptosis inhibition by SAR of Tozasertib analogues. **a** Structure of Tozasertib and an illustration of the four different positions that have been altered to the molecule resulting in 67 Tozasertib analogues (aliphatic substituents (R1), pyrazole moieties (R2), cyclohexyl moieties (R3) and S<>N linker (X)). **b–c** The 67 Tozasertib analogues were screened for both cytokinesis defects and inhibition of necroptosis in one cellular screening assay. Scatter plots of cell growth (amount of nuclei/frame) or nuclear area in relation to necroptosis-inhibiting capacity are shown. L929sAhFas cells were pre-treated with Tozasertib or its analogues (3 µM) for 0.5 h or 24 h. Next, cells were left unstimulated or stimulated with mTNF (40 ng/mL) + zVAD.fmk (10 µM) for 3 h. Proliferation, nuclear area and cell death were quantified by staining the cells with propidium iodide (PI) (3 µM) and Hoechst (1 µM) for image analysis with BD pathway. Percentage of cell death (% PI-positive nuclei) was calculated as percent of control (DMSO). **d** The selection of Tozasertib analogues that inhibited necroptosis was validated in a fluorometric cell death assay over time using SytoxGreen (SG) (5 µM). L929sAhFas cells were pre-treated with Nec1s, Tozasertib or its analogues (3 µM) for 0.5 h, followed by mTNF (20 ng/mL) stimulation. Significance was analysed using repeated measurements data analysis. Data represent mean values ±S.E.M. (*n* = 4). * refers to significance compared to DMSO, ♦ refers to significance compared to Tozasertib. **e** Ranking of validated Tozasertib analogues according to their potential to inhibit necroptosis. Nec1s, Tozasertib or its analogues (3 µM) were ranked for two conditions in L929sAhFas cells: mTNF (20 ng/mL) and mTNF (20 ng/mL) + zVAD.fmk (10 µM)-induced necroptosis, the latter being a sensitising condition for induction of necroptosis. Cells were stimulated for 7 h with mTNF and 4 h with mTNF + zVAD.fmk. Data represent mean values ±S.E.M. (*n* = 2). One-way ANOVA was performed using Tukey correction for multiple comparison (compounds compared to both DMSO control (*) and Tozasertib (♦)). **f** Heat map of screening data (**b**–**c**). Dose responses were included in the screening (**b–c**) assay conditions. IC50 values were determined for cell growth (amount of nuclei/frame), nuclear area and necroptosis protection after 0.5 h or 24 h pre-treated with compound. Hierarchical clustering (row dendrogram using Ward’s hierarchical clustering method with normalisation using *Z*-score calculation) of the IC50 values was then applied on these results in order to create phenotypic clusters and allow SAR analysis of Tozasertib and its analogues (Tibco Spotfire software). Selected analogues for further characterisation are indicated with arrows on the heat map

In order to analyse the required chemical structures that define induction of cytokinesis and necroptosis inhibition in more detail and to design therapeutic Tozasertib analogues that would target RIPK1 with high specificity and potency, 67 Tozasertib analogues were developed (Hofmans S. et al., manuscript under review) and tested for both cytokinesis and necroptosis inhibition (PI positivity with both 0.5 h and 24 h pre-treatment) (Fig. 2). These analogues were designed by changing 4 different positions of Tozasertib (Fig. 2a, Hofmans S. et al., manuscript under review). Scatter plots of cell growth or nuclear area in relation to the necroptosis-inhibiting capacity indicate that Tozasertib inhibits both cellular processes (Fig. 2b, c). Several Tozasertib analogues lose their potency to inhibit these cellular processes, while others preferably still inhibit cytokinesis and proliferation without affecting necroptosis (UAMC2550 and UAMC3033), or vice versa (UAMC3062, UAMC3063 and UAMC3064) (Fig. 2b, c). Also, the Aurora A inhibitor MLN-8054 was included in the screening as benchmark of cytokinesis inhibition without affecting RIPK1-dependent cell death (Fig. 2b, c). These data confirm the previous results (Table 2) that Aurora kinase inhibition has no contribution to the necroptosis inhibition. All necroptosis inhibitors have been validated using a kinetic cell death assay (Fig. 2d)^33^. Both after mTNF and mTNF+zVAD.fmk (sensitising condition) stimulation in L929sAhFas cells, a similar ranking of the Tozasertib analogues was obtained, with UAMC3062, UAMC3063 and UAMC3064 being more potent necroptosis inhibitors than Tozasertib (Fig. 2d, e). Although analogues were identified with better specificity to necroptosis inhibition compared to Tozasertib, they were not more efficient than Nec1s (Fig. 2d, e). Next, hierarchical clustering of all analogues using cytokinesis and cell death IC50 values resulted in the identification of different classes of Tozasertib analogues (Fig. 2f). This Ward’s method clustering identified analogues with increased selectivity for one or the other phenotype (Fig. 2f). Compared to Tozasertib, the analogues UAMC3062, UAMC3063 and UAMC3064 were more selective for necroptosis protection and UAMC3033 and UAMC2550 were more selective for cytokinesis defects (Fig. 2f and Supplementary Figure 4). Overall, based on the characterisation of the cellular phenotypes, analogues were identified with higher selectivity to either Aurora kinases or RIPK1 but not higher potency compared to Tozasertib and Nec1s, respectively.

### Potency and specificity ranking of Tozasertib analogues is confirmed by in vitro kinase activity profiling and validated in a human and mouse cell line

**Fig. 3:**
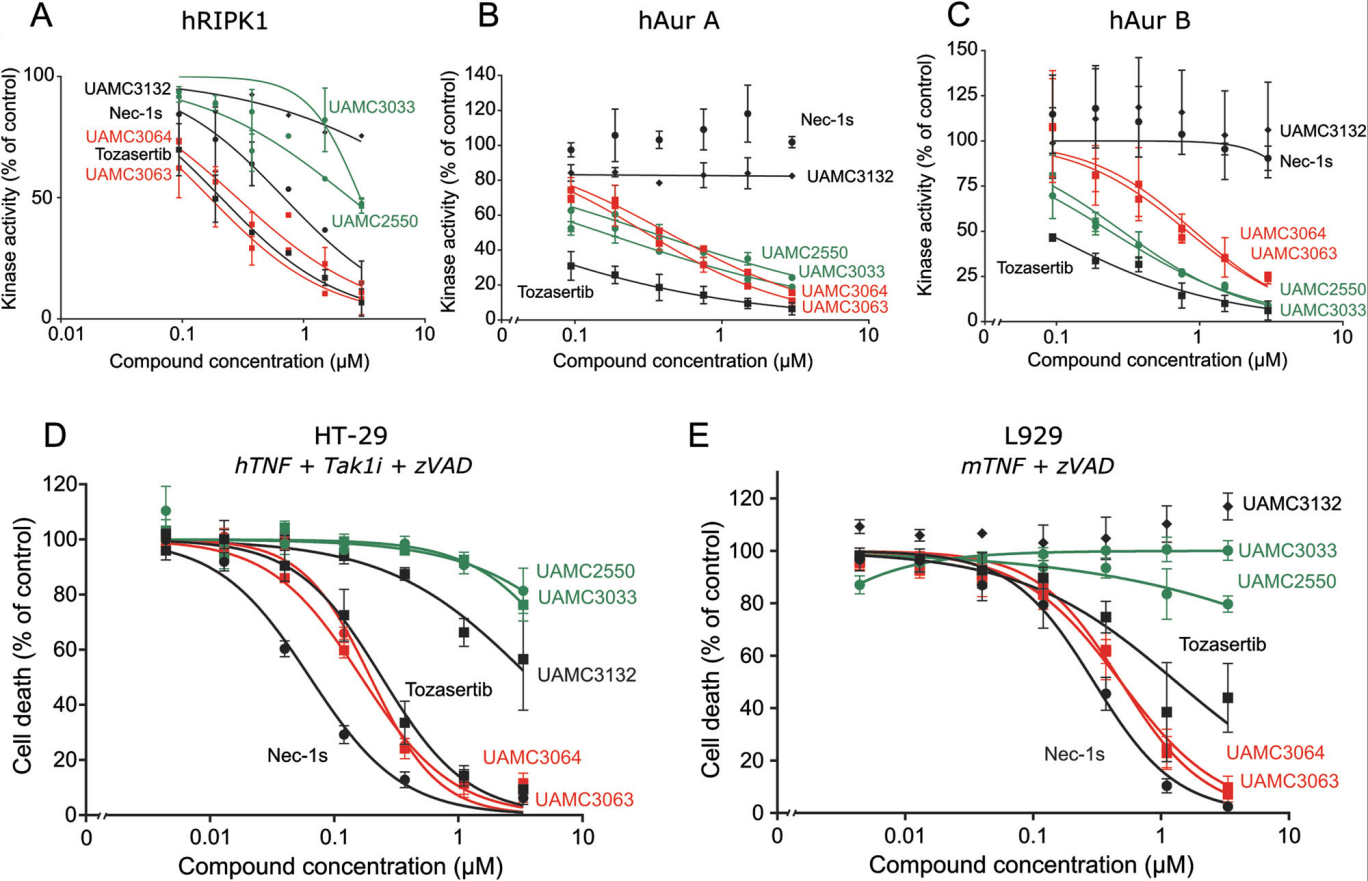
Potency and specificity ranking of Tozasertib analogues is confirmed by in vitro kinase activity profiling and validated in a human and mouse cell line. **a–c** An in vitro ADP-Glo kinase assay using recombinant human (h)RIPK1 (100 nM) (**a**), hAur A kinase (25 nM) (**b**) or hAur B kinase (25 nM) (**c**) was performed. Recombinant protein was incubated with the selected analogues, according to potency and specificity (selection indicated), at concentrations as indicated. Also, Nec1s and Tozasertib were included. Data represent mean value ±S.E.M. (*n* = 2). **d** Human HT-29 cells were pre-treated with Nec1s, Tozasertib and the Tozasertib analogues (concentration as indicated) for 1 h, followed by stimulation with hTNF (100 ng/mL) + Tak1i (1 µM) + zVAD.fmk (20 µM) for 17 h. Cells were stained with SytoxGreen (5 µM) and cell death was measured using the FluoStar fluorescence detection system. **e** Murine L929sAhFas cells were pre-treated with Nec1s, Tozasertib and the Tozasertib analogues (concentration as indicated) for 1 h, followed by stimulation with mTNF (20 ng/mL) for 3 h. Percentage cell death (% PI-positive nuclei) was determined as percent of control. **d–e** Data represent mean value ±S.E.M. (*n* = 3)

Since all data so far suggested that Tozasertib is a RIPK1 inhibitor and Tozasertib was shown to bind to RIPK1^17^, we performed ADP-Glo kinase activity assays using recombinant hRIPK1, hAurora A (hAur A) and hAurora B (hAur B) (Fig. 3a–c). In order to confirm specificity, IC50 values were determined for all three recombinant kinases. Indeed, Tozasertib inhibits hAur A and hAur B kinase activity with an IC50 value of 0.030 µM and 0.068 µM, respectively, but it also inhibits hRIPK1 kinase activity with an IC50 of 0.18 µM (Fig. 3a–c and Supplementary Table 1). Inhibition of necroptosis by Tozasertib seems to act specifically through RIPK1 inhibition, since Tozasertib is not able to inhibit recombinant mRIPK3 kinase activity (Supplementary Figure 5). Although IC50 values for the cellular phenotypes were similar, the IC50 values for inhibition of recombinant kinase activity differs between the Aurora kinases and RIPK1 (Table 1 and Fig. 3a–c). The analogues that showed proliferation and cytokinesis defects without inhibition of necroptosis (UAMC3033 and UAMC2550) inhibit hAur A and hAur B kinase activity, albeit with lower potency than Tozasertib (IC50s between 0.2 and 0.4 µM), whereas limited inhibition of hRIPK1 kinase activity is observed (Fig. 3a–c and Supplementary Table 1). On the other hand, the analogues with higher specificity for necroptosis inhibition compared to Tozasertib (UAMC3063 and UAMC3064) could inhibit hRIPK1 kinase activity with a similar dose response as Tozasertib. Although no cytokinesis and proliferation defects were detected based on the cellular parameters analysed, these analogues are still able to inhibit hAur A and hAur B kinase activity, albeit with a >10-times lower potency than Tozasertib (Fig. 3a–c and Supplementary Table 1). Due to redundancy in the function of Aurora A and B, loss in potency of both Aurora A and B inhibition, may give rise to the more specific cell death phenotype for these analogues. Also, for Aurora kinase inhibition, IC50 values between recombinant kinase assays and cellular phenotypes differ significantly from nanomolar range for the recombinant kinase assays to micromolar range for cellular phenotypes (Table 1 and Supplementary Table 1). This may be due to timing and parameter chosen for the cellular assay, or to poor membrane permeability. Therefore, small changes in the enzymatic IC50 values could result in significant changes in cellular phenotype. Nec1s only inhibits recombinant hRIPK1 and the inactive analogue UAMC3132 lost the ability to inhibit all three recombinant proteins (Fig. 3a–c). Overall, these data indicate that the inhibition of Aurora kinases and RIPK1 by Tozasertib and its analogues is correlated to the proliferation and cytokinesis defects and necroptosis protection, respectively.

In a final step, the selected analogues with increased specificity were also tested in a cell death assay in the human HT29 (Fig. 3d) and the mouse L929sAhFas (Fig. 3e) cell lines (sensitised condition). After stimulation with hTNF+Tak1i+zVAD.fmk to induce necroptosis in HT29, UAMC3063-UAMC3064 could protect with similar IC50 values as Tozasertib (0.1–0.2 µM), while UAMC2550-UAMC3033 (more specific for Aurora kinase) and the inactive analogue UAMC3132 could not protect (Fig. 3d and Supplementary Table 2). Similar results were obtained in mouse L929sAhFas cells stimulated with mTNF+zVAD.fmk, a sensitising necroptotic condition (Fig. 3e and Supplementary Table 2). Overall, these data illustrate that Tozasertib targets RIPK1, but not RIPK3, and that Tozasertib analogues were designed with increased specificity for Aurora kinase or RIPK1 as characterised with human and mouse cellular phenotypes and with direct inhibition of recombinant kinases.

## Discussion

Tozasertib, also known as VX-680^34^, is a small molecule type I kinase inhibitor targeting the Aurora family of serine/threonine protein kinases^15^. This kinase family is crucial for proper proliferation and cell cycle progression^3,4,7,35^. Increased expression or gene amplification of Aurora A/B has been observed in a number of cancers^6,8,36,37^ and its oncogenic potential has been illustrated by the fact that Aurora A overexpression induces transformation of mammalian fibroblasts^38^. Tozasertib-induced inhibition of Aurora kinase activity results in cell proliferation defects, disruption of bipolar spindle formation, polyploidy and finally cell death^3,6,39–43^. These phenotypic parameters are associated with defective mitosis and cytokinesis. Tozasertib has entered phase I/II clinical trials for the treatment of solid tumours^44^, chronic myeloid leukaemia and acute lymphocytic leukaemia with T351I BCR-ABL mutations^45–47^. Although Tozasertib treatment has clear anti-tumour activity, studies were discontinued due to toxic adverse effects including febrile neutropenia, anaemia and thrombocytopenia^5,16^. Type I kinase inhibitors are known to have more than one possible target or binding partner. Also, Tozasertib has been shown to bind other kinases than Aurora. Other kinase targets that have been described for Tozasertib are the oncogenic kinases Flt-3 and Abl (both wild-type Abl kinase and imatinib-resistant Abl mutant (T315I)) and JAK 2 kinase, with the latter playing a role in imatinib resistance in chronic myelogenous leukaemia^3,5,14,15,45,48^. Davis et al. performed an interaction study consisting of 72 kinase inhibitors and 442 kinases, covering more than 80% of the human catalytic protein kinome^17^. This study showed that Tozasertib binds to RIPK1 with a *K*_d_ of 20 nM, suggesting that Tozasertib could inhibit TNF-induced necroptosis, a RIPK1-dependent pathway^19,21,49,50^. In our study, we show that Tozasertib not only targets Aurora kinases, resulting in proliferation and cytokinesis defects, but also inhibits TNF-induced necroptosis and that its potency is similar for both cellular phenotypes. Both in murine (L929sAhFas, MEF cells) and human (HT-29) cell lines, Tozasertib inhibits TNF-induced necroptosis at concentrations below 10 µM (IC50 HT-29 is 0.3 µM and IC50 L929 is 0.7 µM). Targeting the active site of kinases, like the competition of Tozasertib with ATP for binding to the active site^14,51^, remains a challenge due to the fact that ATP-binding pockets are very similar between kinases^14^. This specificity issue for kinase inhibitors is a.o. illustrated by a recent report about PERK inhibitors that are also very potent RIPK1 inhibitors^52^. Even when sequence identity with Aurora kinase is less than 50%, which is the case for Flt3 and Abl, kinase inhibition by Tozasertib is still observed^3^, indicating that other factors (a.o. structural properties) may also influence interaction of the kinases with Tozasertib.

In order to study the possible relation between Aurora kinase inhibition and RIPK1-dependent necroptosis inhibition, other known Aurora kinase inhibitors (AMG-900, Barasertib, Danusertib, SNS-314 mesylate) were tested for inhibition of TNF-induced necroptosis. Except for SNS-314 mesylate, these Aurora kinase inhibitors were not able to protect against TNF-induced necroptosis, illustrating that Aurora and necroptosis inhibition are independent processes. Nec1 and Nec1s were the first RIPK1 inhibitors discovered and are now used in research as benchmark for RIPK1 inhibition^19,49,53,54^. Other necrostatins have also been developed^49,55–57^, but although they had proper selectivity, pharmacokinetic properties of all necrostatins were moderate^58^. This indicates the need for better therapeutic RIPK1 inhibitors. Recently, GSK developed a type II/III RIPK1 inhibitor (GSK2982772) that is currently in clinical trial phase IIa for the treatment of psoriasis, rheumatoid arthritis and ulcerative colitis^59,60^. Since necrostatins and the GSK RIPK1 inhibitor are classified as type II or III kinase inhibitors, and Tozasertib has structural elements of a type I kinase inhibitor (targeting the ATP-binding pocket of the kinase), this created the opportunity to develop type I RIPK1 inhibitors based on Tozasertib structure. Therefore, 67 Tozasertib analogues were developed (Hofmans S. et al, manuscript under review) and tested for cytokinesis defects and necroptosis protection in order to design novel type I RIPK1 inhibitors with higher potency and selectivity than Tozasertib. In a second step, analogues that inhibited necroptosis were validated using a real-time fluorometric method^33^. After detailed analysis and clustering of IC50 values, Tozasertib analogues were selected that had increased specificity for either cytokinesis defects (UAMC3033 and UAMC2550) or necroptosis protection (UAMC3063 and UAMC3064). These selected Tozasertib analogues have been tested for their potency to inhibit recombinant kinases Aur A, Aur B and RIPK1. These data confirmed our observation in the cellular assay that the selected analogues have similar potency for Aurora and/or RIPK1, but show increased specificity. Although analogues specific for necroptosis (RIPK1) inhibition in cellular assays still inhibited recombinant Aurora kinases, the more than tenfold decrease in potency is enough to reduce significantly the cytokinesis defects in the cellular assay.

It is not clear how Tozasertib and its analogues bind RIPK1. Each of the Tozasertib analogues can have a very different binding and interaction with both Aurora A, B and RIPK1. Indeed, kinases can have two different conformations, the ‘DFG-in’ active conformation and the ‘DFG-out’ inactive conformation^61^. RIPK1 has a DLG motif instead of the well-known DFG motif, resulting in different organisation of the amino acids during catalysis. Necrostatins (Nec-1a, Nec-3a and Nec-4) can stabilise RIPK1 in an inactive conformation that has structural similarities with the inactive conformation of the protein PKA (with DFG motif)^55^. Many therapeutic kinase inhibitors bind the closed conformation (Imatinib, GW572016 and A-770041) by making hydrophobic interactions with the enzyme^14,62–64^, but Tozasertib has a different way to interact with Aurora kinases. Tozasertib is described as a type I kinase inhibitor that binds at the ATP site without penetrating the allosteric pocket and therefore does not depend on specific kinase conformation for its binding^65,66^. Full activation of Aurora A kinase is only achieved when it binds to its co-factor targeting protein for Xklp2 (TPX2). Binding of TPX2 causes the activation loop to fold away from the active site, providing the DFG-in ‘open’, active kinase conformation^67,68^. Similar interaction of Aurora B with its co-factor inner centromere protein (INCENP) has been described^69^. Depending on the presence of the co-factor, Tozasertib can bind different conformations. Tozasertib was shown to bind to the closed conformation of Aurora A in the absence of TPX2^14,70,71^. On the other hand, in the presence of TPX2 it binds to the open, active conformation of Aurora A^51,67^. Tozasertib also binds to the DFG-in ‘open’, active conformation of the imatinib-resistant form of Abl kinase containing H396P mutation^65^. This shows that prediction of the binding mode of Tozasertib, and especially its analogues, to RIPK1 (and Aurora kinase) will be difficult due to the high flexibility of these kinases (switching between inactive–active conformations) and needs further investigation.

Tozasertib is able to reduce tumour growth in vivo in xenograft models of leukaemia and colon cancer (HL-60 and HCT-116)^15^. With its clear anti-cancer activity^15,39,45^ and apoptosis-inducing capacity^42,43^, it is surprising that it at the same time inhibits TNF-induced necroptotic cell death at concentrations below 10 µM. But, Tozasertib is not the only chemotherapeutic agent that inhibits necroptosis. Other kinase inhibitors have been described to inhibit RIPK1 and/or RIPK3: ponatinib^72,73^, dabrafenib^74^, pazopanib^72^ and Sorafenib^75^. The fact that so many chemotherapeutic kinase inhibitors have been selected and turned out to have necroptosis inhibition properties may suggest that necroptosis targeting paradoxically may be part of the anti-tumour mechanism. It could also be that it just reflects the high chance for resemblance of ATP pocket targeting kinase inhibitors, especially small molecule type I inhibitors designed to insert into the ATP pocket, a feature that can have high similarity among kinases. However, the idea that proteins of the core pathway of necroptotic cell death (RIPK1/3 and MLKL) can promote tumour growth has been recently published^22,23,76,77^. During necroptosis, the cell explodes and releases damage-associated molecular patterns (DAMPs)^78^. These events may happen in the necrotic core^22^. This continuous presence of necrotic dying cells and the released DAMPs could create a pro-tumorigenic inflammatory environment that enhances the growth of the tumour mass. This could explain why targeting RIPK1 could contribute to the anti-cancer activities of these chemotherapeutics. Overall, we show that Tozasertib directly targets RIPK1 and therefore protects against TNF-induced necroptosis, and that Tozasertib analogues were designed with increased specificity for Aurora kinases or RIPK1 kinase.

## Materials and methods

### Cell culture, cytokines and reagents

L929 cells stably transfected with hFas and designated as L929sAhFas were generated as previously described^30^. All L929 cell lines and MEF cells were cultured in DMEM supplemented with 10% (v/v) FCS and l-glutamine (0.03%). HT-29 cells were cultured in EMEM medium supplemented with 10% (v/v) FCS and l-glutamine (0.03%). Following cytokines were used: recombinant (rec) human and murine TNF-α were produced at VIB protein Service Facility (Ghent, Belgium) with a specific biological activity of 3 × 10^7^ IU/mg and 1.27 × 10^8^ IU/mL, respectively. Following reagents were purchased as indicated: Nec1s (synthesised by the Laboratory of Medicinal Chemistry; University of Antwerp), GSK’963 (Aobious, AOB9775, Gloucester, MA), Tozasertib (LC Laboratories, T-2304), Tozasertib analogues (synthesised by the Laboratory of Medicinal Chemistry; University of Antwerp), Tak1 kinase inhibitor NP-009245 (AnalytiCon Discovery GmbH, Potsdam, Germany), z-VAD-fmk (Bachem, Bubendorf, Switzerland and R&D systems) and agonistic anti-human Fas (clone 2R2, Cell Diagnostica, Munster, Germany).

### Antibodies and immunoblotting

Antibodies used were anti-phospho-Aurora A (Thr288)/Aurora B (Thr232)/Aurora C (Thr198) (D13A11) (Cell Signaling, #2914, Danvers, MA, USA), anti-tubulin-HRP (abcam, ab21058, Cambridge, UK). After treatments, cells were washed twice with PBS and lysed with 2× Laemmli buffer directly. These samples were used for SDS-PAGE and immunoblotting. Tubulin was used as loading control.

### Growth arrest and cell death analysis

Proliferation, cytokinesis defects and cell death were analysed using a BDPathway 855 high-content screening instrument (BD Biosciences)^31,79^. Ten thousand (1 h assay) or five thousand (24 h assay) cells were seeded in a black-clear 96-well plate. The next day, the cells were pre-treated with the indicated compounds for 1 or 24 h and then stimulated with mTNF, hTNF or agonistic anti-Fas Ab (concentration as indicated) in the presence of 3 µM propidium iodide (Sigma-Aldrich) and 1 µM Hoechst (Sigma-Aldrich). Per condition, 25 image frames were taken with a ×20 objective containing a minimum of 1000 cells. Data were analysed using the Columbus software package (PerkinElmer). The image algorithm identifies objects as nuclei using the Hoechst channel and categorises cells as life or dead using the PI channel. The percentage of PI+ nuclei is calculated as read-out for cell death. The total number of nuclei after 24 or 48 h treatment reflects cell growth and nuclear area/nuclear roundness are the parameters that measure Aurora kinase inhibition and therefore growth arrest and cytokinesis defects. For time kinetics and when indicated, cell death was analysed on a FLUOSTAR Omega (BMG Labtech, Offenburg, Germany). Cells were stained with 5 µM SytoxGreen (Life Technologies) and 33 µM ac-DEVD-amc (PeptaNova GmbH, Sandhausen, Germany) together with treatment as indicated. Maximal cell death was obtained by permeabilizing all cells with Triton X-100 (0.05%). Cell death and caspase activity was determined as described previously^33^.

### Clonogenic assay

L929sAhFas cells were treated with Tozasertib (concentration as indicated) for 24 h. Next, treatment was removed and 50 cells/condition were seeded. After 10 days, cells were fixed with 4% PFA for 10 min. After fixation, cells were stained with a 0.01% crystal violet solution for 1 h. Cells were washed with PBS, dried overnight and images were taken. Colonies were counted using ImageJ.

### In vitro ADP-Glo kinase assay

Compounds (concentrations as indicated) were co-incubated with recombinant hRIPK1 (100 nM) (produced in our laboratory), hAurora kinase A (25 nM) or hAurora kinase B (25 nM) (ADP-Glo^TM^ kinase assay + Aurora A/Aurora B kinase enzyme system, Promega, V9081 and V9181). For hRIPK1, the kinase assay buffer (2×) contains 50 mM HEPES pH 7.5, 30 mM MgCl_2_, 50 mM NaCl, 0.5 mg/mL BSA, 0.02% CHAPS and 1 mM DTT. For the Aurora kinases, the kinase assay buffer was used from the promega assay (V9081, V9181). Next, the in vitro ADP-Glo^TM^ kinase assay (Promega, V6930) was used to determine kinase activity. The assay was performed according to the manufacturer’s protocol. The primary kinase reaction was carried out for 4 h at room temperature and a 2:2:1 ratio of kinase reaction volume to ADP-Glo reagent volume to kinase detection reagent volume was used.

### Statistics

Two or three independent experiments were performed (as indicated in Figure legends) and data were analysed using GraphPad Prism 7 (when indicated). Values represent the mean values ± standard error of the mean, unless indicated otherwise. IC50 values were calculated using nonlinear curve fitting (four parameters) with fixed top and bottom in GraphPad Prism 7 (Table 2). All parameters, in which the compounds are inhibitory, are indicated with IC50 values. Only for nuclear area, the values indicate EC50, as Tozasertib (and analogues) enhances nuclear area. The definition of IC50 and EC50 is also indicated in the legends of the Tables. When indicated, one-way ANOVA was performed using Tukey correction for multiple comparison. *p < 0.05, **p < 0.01, ***p < 0.001, ****p < 0.0001, with *, Δ and ♦ referring to significance compared to respectively DMSO control, Nec-1s and Tozasertib.

#### Repeated measurements data analysis (FIG1G and FIG2D)

Percentages of cell death were analysed as repeated measurements using the residual maximum likelihood (REML) approach as implemented in Genstat v18 (Genstat Reference Manual (Release 18), Part 3 Procedures. 2015. VSN International, Hemel Hempstead, UK). Briefly, a linear mixed model with compound, time and compound × time interaction as fixed terms, and subject.time used as residual term, was fitted to data. Times of measurement were set at equal intervals and an autoregressive correlation structure of order 1 (AR1) with equal variances across time, was selected as best model fit in all cases, based on the Aikake information coefficient. Significances of the fixed terms and pairwise comparisons of compounds across time were assessed by an F-test.

#### Probit analysis

Probit analysis, as implemented in Genstatv18 (Genstat Reference Manual (Release 18), Part 3 Procedures. 2015. VSN International, Hemel Hempstead, UK), was used to model the relationship between the doses of compounds and the cell death response or kinase activity. Doses were transformed to logarithms and IC50 values for each compound were calculated. The dispersion parameter was fixed to 1 and used when calculating standard errors of the IC50 values. The significance of differences between EC50 values was assessed on a log-scale using a *t* test, before back transforming to the original scale.

## Electronic supplementary material

supplementary figures

Legends supplementary figures
