# Supplementary material for: RIPK1-dependent cell death: a novel target of the Aurora kinase inhibitor Tozasertib (VX-680)
Source: Cell Death Dis. 2018 Feb 12;9(2):211. doi: 10.1038/s41419-017-0245-7 (PMC5833749; doi:10.1038/s41419-017-0245-7)

Supplementary figure 1

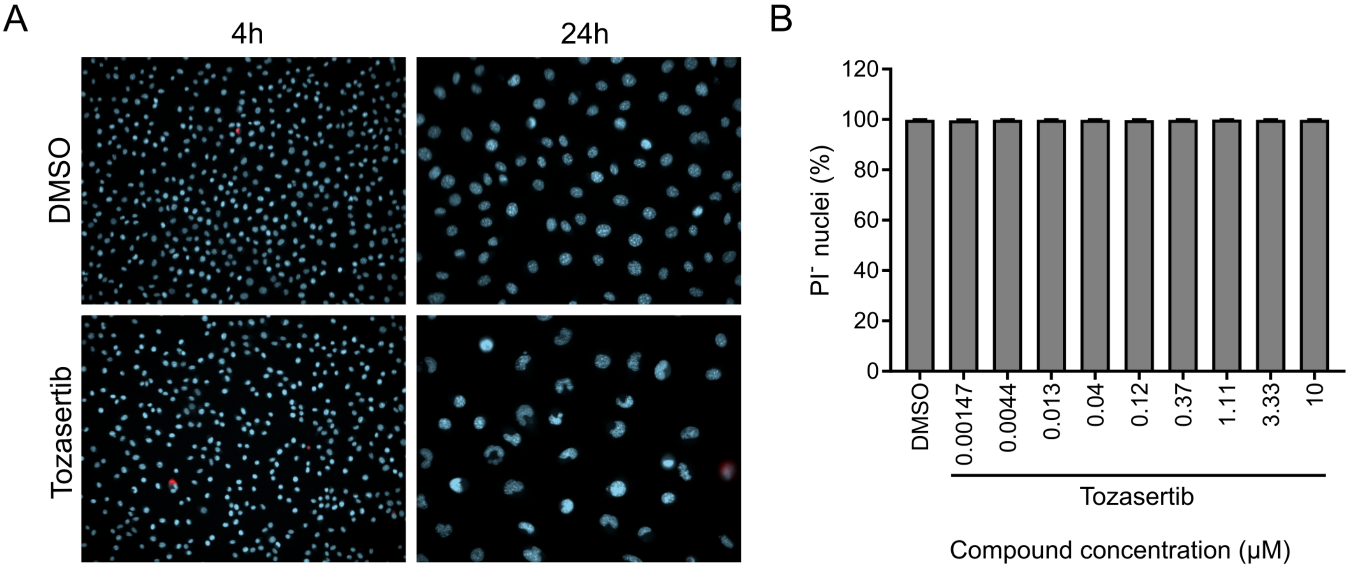

Supplementary figure 2

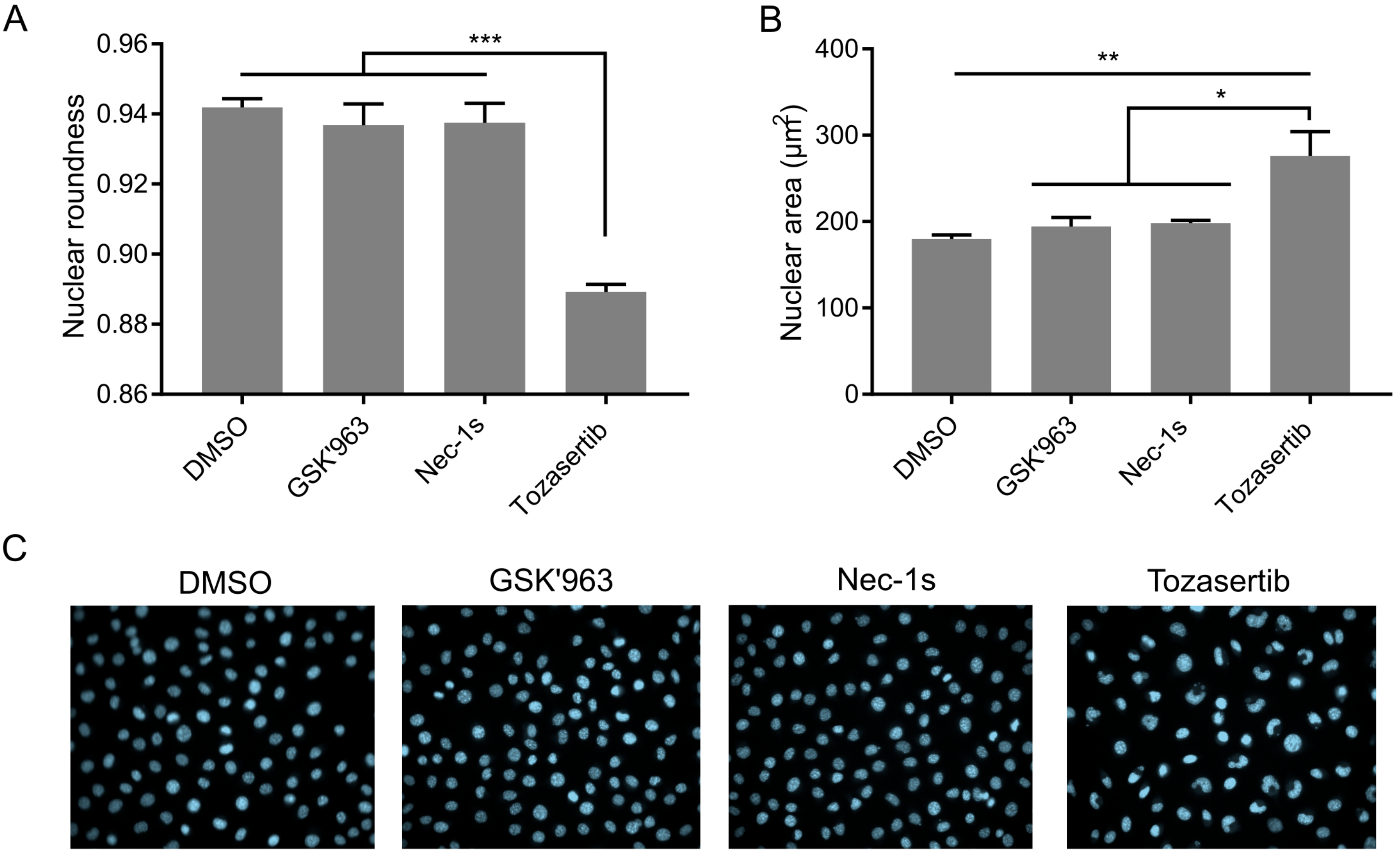

Supplementary figure 3

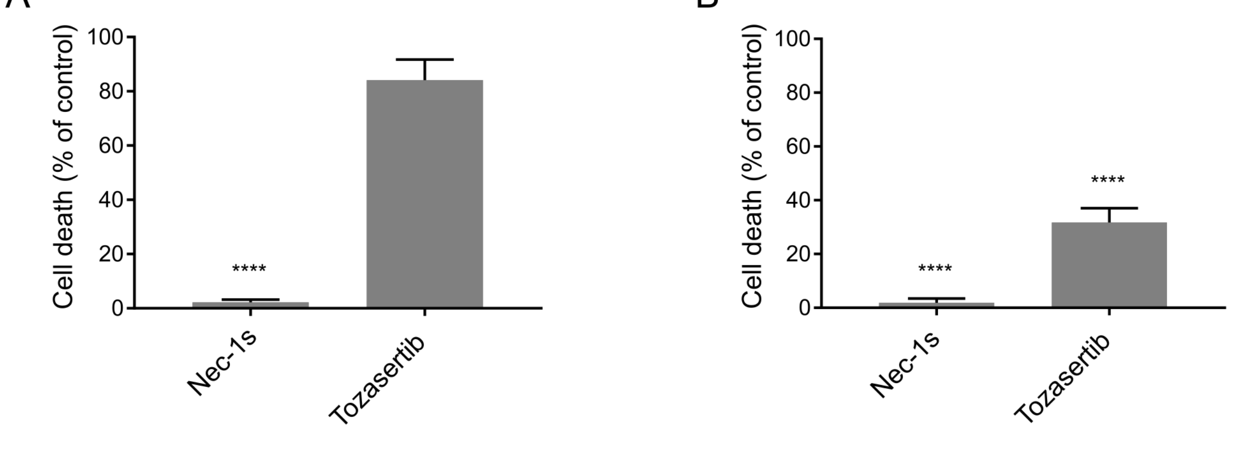

Supplementary table 1

	hRIPK1		hAur A		hAur B	
	**IC50**	**95% CI for IC50**	**IC50**	**95% CI for IC50**	**IC50**	**95% CI for IC50**
**Nec-1s**	0.754	0.641 to 0.888	NC	NC	NC	NC
**Tozasertib**	0.208[ΔΔΔ]	0.175 to 0.247	0.030	0.023 to 0.040	0.068	0.056 to 0.082
**UAMC3132**	NC	NC	NC	NC	NC	NC
**UAMC3033**	NC	NC	0.370♦♦♦	0.303 to 0.450	0.274♦♦♦	0.228 to 0.328
**UAMC2550**	2.468[ΔΔΔ]	2.030 to 3.030	0.210♦♦♦	0.172 to 0.260	0.229♦♦♦	0.193 to 0.271
**UAMC3063**	0.167[ΔΔΔ]	0.139 to 0.199	0.430♦♦♦	0.350 to 0.530	0.853♦♦♦	0.710 to 1.028
**UAMC3064**	0.295[ΔΔΔ]	0.249 to 0.347	0.310♦♦♦	0.253 to 0.380	0.959♦♦♦	0.794 to 1.162

Supplementary table 2

	**HT29** (hTNF+zVAD+Tak1i)		**L929** (mTNF+zVAD)	
	**IC50**	**95% CI for IC50**	**IC50**	**95% CI for IC50**
**Nec-1s**	0.073♦♦♦	0.063 to 0.084	0.247♦♦♦	0.210 to 0.291
**Tozasertib**	0.219	0.189 to 0.253	1.366	1.138 to 1.646
**UAMC3132**	NC	NC	NC	NC
**UAMC3033**	NC	NC	NC	NC
**UAMC2550**	NC	NC	NC	NC
**UAMC3063**	0.202	0.174 to 0.235	0.518♦♦♦	0.439 to 0.612
**UAMC3064**	0.175	0.151 to 0.202	0.486♦♦♦	0.413 to 0.573

Supplementary figure 4

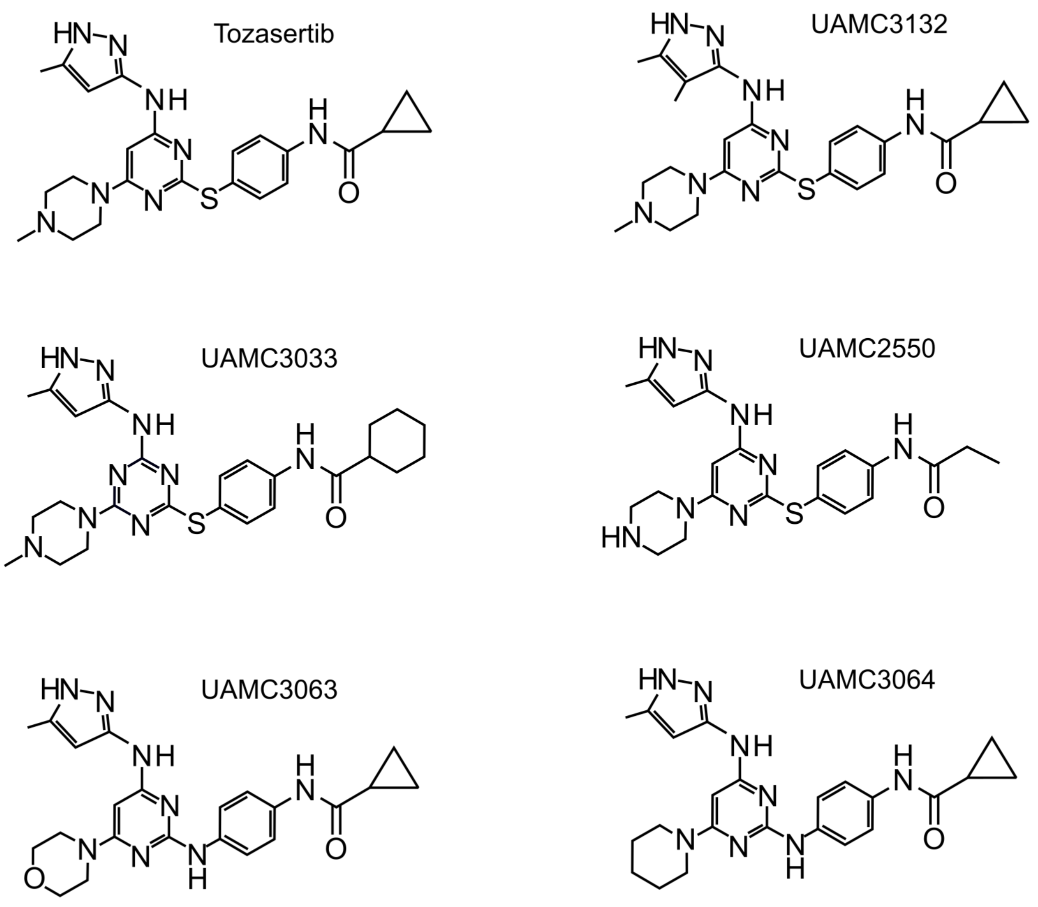

Supplementary figure 5

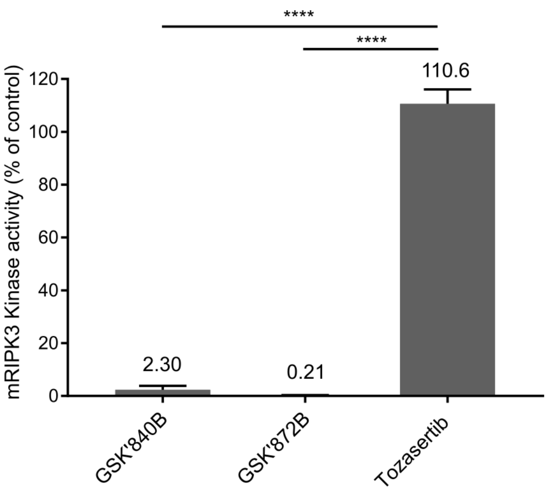

Supplement: Supplementary file 1 — supplementary figures [file 41419_2017_245_MOESM1_ESM.pdf]
