# Supplementary material for: RIPK1-dependent cell death: a novel target of the Aurora kinase inhibitor Tozasertib (VX-680)
Source: Cell Death Dis. 2018 Feb 12;9(2):211. doi: 10.1038/s41419-017-0245-7 (PMC5833749; doi:10.1038/s41419-017-0245-7)
Supplement: Supplementary file 2 — Legends supplementary figures [file 41419_2017_245_MOESM2_ESM.doc]

**Supplementary Figure and Table Legends**

**Supplementary Figure 1. Tozasertib is not cytotoxic in L929sAhFas cells.** L929sAhFas cells were treated with 10 µM Tozasertib for 4h or 24h (A) or with Tozasertib (concentration as indicated) for 24h (B). Cells were stained with Hoechst (1 µM) and propidium iodide (PI) (3 µM) and high-content images were acquired using BD pathway Bio-imager (A) and analysed using Columbus^TM^ software (B). Live cells (% PI-negative cells) are quantified for each concentration of Tozasertib (B). Data represent mean ± S.E.M. of 3 independent experiment. One-way Anova with Tukey correction for multiple testing has been performed.

**Supplementary Figure 2. RIPK1 inhibition does not affect cytokinesis.** L929sAhFas cells were treated with Tozasertib, RIPK1 inhibitor GSK’963 or RIPK1 inhibitor Nec1s (10 µM) for 24h. Cells were stained with Hoechst (1 µM) and propidium iodide (PI) (3 µM) and high-content images were acquired using BD pathway Bio-imager (C) and analysed using Columbus^TM^ software (A-B). Both nuclear roundness (A) and nuclear area (B) was determined for all conditions, with nuclear roundness quantified as 0 being a straight line and 1 being a perfect circle. Data represent mean ± S.E.M. (n=3). One-way Anova with Tukey correction for multiple testing has been performed. Inhibitors are compared to Tozasertib (*p<0.05 , **p<0.01 , ***p<0.001).

**Supplementary Figure 3. Tozasertib partially loses protective effect against necroptosis, only in sensitizing condition in L929sAhFas cells and in human HT-29 cells at 10 µM.** L929sAhFas cells were pre-treated with DMSO, Tozasertib or Nec1s (10 µM) for 1h, followed by stimulation with mTNF (20 ng/mL) + zVAD.fmk (20 µM) for 3h (A). HT-29 cells were pre-treated with DMSO, Nec1s or Tozasertib (10 µM) for 1h, followed by stimulation with hTNF (100 ng/mL) + Tak1i (1 µM) + zVAD.fmk (20 µM) for 16h (B). Cell death % (percent of control) was determined based on PI staining (3 µM) (A) or SytoxGreen staining (5 µM) (B). (A-B) Data represent mean ± S.E.M. of 3 independent experiment. One-way ANOVA with Tukey correction for multiple testing has been performed, comparing Nec1s and Tozasertib to DMSO (100%) (****p<0.0001).

**Supplementary Figure 4. Structure of the selected Tozasertib analogues.** Structures of Tozasertib, UAMC3132 (lost activity against both RIPK1 and Aurora kinase), UAMC3033 and UAMC2550 (analogues more specific for Aurora kinase), UAMC3063 and UAMC3064 (analogues more specific for RIPK1 kinase).

**Supplementary Figure 5. Tozasertib does not inhibit recombinant mRIPK3 kinase activity.** An *in vitro* ADP-Glo kinase assay using recombinant mRIPK3 (15 nM) (A) was performed. Recombinant protein was incubated with Tozasertib, RIPK3 inhibitor GSK’840B and GSK’872B (10 µM). Data represent mean value ± S.E.M. of 3 independent experiment. One-way ANOVA with Tukey correction for multiple testing has been performed (**** p<0.0001 ).

**Supplementary Table 1. IC50 determination of *in vitro* kinase assays with hRIPK1, hAurora A and hAurora B.** Table of IC50 values corresponding to Figure 3A-C, where Nec1s, Tozasertib and the selected analogues are tested for inhibition of kinase activity of recombinant hRIPK1, hAurora A and hAurora B. IC50 values were calculated and compared using Probit analysis (see material and methods). Δ refers to the significance of the difference relative to the IC50 of Nec1s,♦ refers to the significance of the difference relative to the IC50 of Tozasertib.The 95% confidence intervals (CI) for IC50s are also indicated. NC = not calculable.

**Supplementary Table 2. Tozasertib and its analogues UAMC3063 and UAMC3064 protect against necroptosis in HT29 and L929sAhFas cells, but the analogues UAMC3132, UAMC3033 and UAMC2550 do not.** Table of IC50 values corresponding to Figure 3D-E, where Nec1s, Tozasertib and the selected analogues are tested for inhibition RIPK1-dependent necroptosis in HT29 and L929sAhFas cells. IC50 values were calculated and compared using Probit analysis (see material and methods). ,♦ refers to the significance of the difference relative to the IC50 of Tozasertib. The 95% confidence intervals (CI) for IC50s are also indicated. NC = not calculable.
